# Achieving High Strength and High Conductivity of Cu-6 wt%Ag Sheets by Controlling the Aging Cooling Rate

**DOI:** 10.3390/ma16103632

**Published:** 2023-05-10

**Authors:** Zhongyuan Zhang, Yadong Ru, Tingting Zuo, Jiangli Xue, Yue Wu, Zhaoshun Gao, Yongsheng Liu, Liye Xiao

**Affiliations:** 1 Institute of Solar Energy, Shanghai University of Electric Power, Shanghai 200090, China; zhangzhongyuan@mail.iee.ac.cn; 2 Institute of Electrical Engineering, Chinese Academy of Sciences, Beijing 100190, China; ruyadong@mail.iee.ac.cn (Y.R.); wuyue@mail.iee.ac.cn (Y.W.); xiao@mail.iee.ac.cn (L.X.); 3 University of Chinese Academy of Sciences, Beijing 100049, China

**Keywords:** high strength-high conductivity, Cu-Ag sheet, aging treatment, cooling rate, Ag precipitated phase

## Abstract

In this paper, Cu-6 wt%Ag alloy sheets were prepared using vacuum induction melting, heat treatment, and cold working rolling. We investigated the influence of the aging cooling rate on the microstructure and properties of Cu-6 wt%Ag alloy sheets. By reducing the cooling rate of the aging treatment, the mechanical properties of the cold-rolled Cu-6 wt%Ag alloy sheets were improved. The cold-rolled Cu-6 wt%Ag alloy sheet achieves a tensile strength of 1003 MPa and an electrical conductivity of 75% IACS (International Annealing Copper Standard), which is superior to the alloy fabricated with other methods. SEM characterization shows that the change in properties of the Cu-6 wt%Ag alloy sheets with the same deformation is due to a precipitation of the nano-Ag phase. The high-performance Cu-Ag sheets are expected to be used as Bitter disks for water-cooled high-field magnets.

## 1. Introduction

Cu-Ag alloys are widely used as conductors for high field magnets due to their high conductivity and high strength, which can reduce Joule heat and resist Lorentz’s force [1,2,3,4,5,6,7]. At present, most water-cooled magnets above 30 T are made of Cu-Ag alloy sheets [8,9,10]. Since Ag is expensive, low Ag content (<8 wt.%) Cu-Ag alloys have been widely studied to reduce the cost [11,12,13,14,15,16,17]. Zhang et al. [18] prepared a Cu-5Ag alloy sheet with a strength of 870 MPa and an electrical conductivity of 78% IACS by obtaining a deformation nanotwin structure at liquid nitrogen temperature. Gaganov et al. increased the tensile strength from 1220 MPa to 1360 MPa by adding the Zr element into Cu-7Ag alloy, but the conductivity of the alloy was significantly reduced from 68% IACS to 58% IACS [19]. An et al. [16] added the Sc element into Cu-6 wt%Ag alloy and the alloy strength increased to 1080 MPa but the conductivity decreased from 77% IACS to 66% IACS. Although researchers have made some breakthroughs by regulating the microstructure of Cu-Ag alloys, low Ag content Cu-Ag alloy sheets with high strength and high conductivity fabricated by simple processing methods have not been realized so far.

Compared with solid solution strengthening, work hardening, fine crystal strengthening, and other strengthening mechanisms, aging strengthening can not only improve the mechanical properties of the alloy but also significantly reduce the resistivity of the copper matrix [20,21,22,23,24,25]. At present, it is known that the precipitated phase obtained by aging treatment has an important effect on the preparation of high-performance Cu-Ag alloys. The aging precipitation of Cu-Ag alloy can be divided into continuous precipitation (CP) and discontinuous precipitation (DP). The main difference between these two precipitations is that the discontinuous precipitated phase nucleates and grows by way of the movement of the grain boundary, while the continuous precipitated phase nucleates and grows within the grain [19,26,27]. In Cu-Ag alloys with low Ag content, there is almost no continuous precipitated phase, only a discontinuous precipitated phase exists at the grain boundary. Compared with the discontinuous precipitated phase, the continuous precipitated phase generated by aging treatment can lead the alloy to high conductivity and higher strength [11,16,19,23,26]. Therefore, the regulation of the precipitated phase in Cu-Ag alloy (inhibition of discontinuous precipitated phase, promotion of continuous precipitated phase) is the key factor in achieving high strength and high conductivity in Cu-Ag alloy with low Ag content.

A large number of studies have shown that aging temperature and time have an important influence on the microstructure and properties of Cu-Ag alloy [26,27,28,29]. The aging cooling process can also affect the precipitation kinetics, such as the diffusion, migration, and precipitation of the solidly dissolved atoms in the alloy [28]. In addition, Cu-Ag alloy is in a high-temperature state for a long time; the cooling rate also affects the coarsening of the precipitated phase. However, all these have not been well understood until now.

In this paper, a Cu-Ag alloy sheet with high strength and great conductivity was prepared using methods of vacuum induction melting, forging, aging treatment, and cold rolling. The influence of the aging cooling rate on the microstructures and properties of Cu-Ag alloys was thoroughly studied.

## 2. Materials and Methods

**Figure 1:**
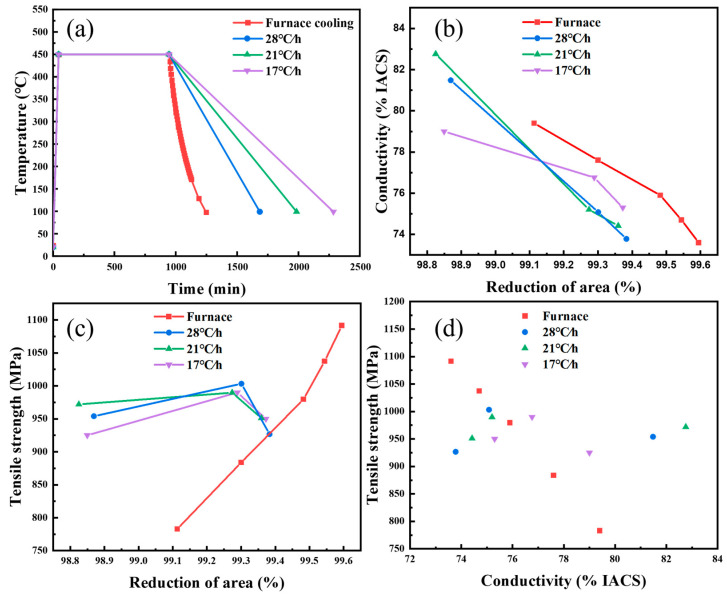
(**a**) Change of aging temperature with time. (**b**) Change of electrical conductivity with the deformation of the Cu-6 wt%Ag alloy sheet. (**c**) Change of strength with the deformation of the Cu-6 wt%Ag alloy sheet. (**d**) Comprehensive performance diagram of the Cu-6 wt%Ag alloy sheet.

The raw materials used were high-purity Ag (99.99% purity) and high-purity Cu (99.99% purity). A Cu-6 wt%Ag ingot with a size of 26 mm× 26 mm× 70 mm was prepared using vacuum induction melting. After melting, the ingot underwent a solution treatment at 780 °C for 2 h, then was quenched into water. Then, the block was deformed using a hydraulic press machine at a deformation of 22.12% and the cross-sectional area went from 26 mm× 26 mm to 23.4 mm× 26 mm. After that, the Cu-Ag alloy was aging-treated at 450 °C for 15 h and cooled at different rates (furnace cooling, 28 °C/h, 21 °C/h, and 17 °C/h), as shown in Figure 1a. Finally, the aged Cu-6 wt%Ag alloy was rolled to a thickness of about 0.3 mm at room temperature. The rolling reduction of the sheet ε% is defined as $100\%\times (t_{0}-t)/t_{0}$, where $t_{0}$ and t are the thickness of the initial sample and that after cold rolling.

Scanning electron microscopy (SEM, Zeiss Sigma, Carl Zeiss AG, Oberkochen, Germany) and energy-dispersive X-ray spectroscopy (EDS) were used to characterize the microstructure. X-ray diffraction (XRD, Bruke D8, Bruker, Karlsruhe, Germany) was also used to characterize the crystallographic integrity of phases after thermomechanical treatment. The electrical conductivity was measured using an eddy current conductivity meter (ECCM, Sigma 2008B, Xiamen Tianyan Instrument Co., Ltd., Xiamen, China). The tensile strength–strain curve of the sample was measured using a universal tensile machine (UTM, CMT6104, Yunnan Xuecu Technology Co., Ltd., Yunnan, China) at a strain rate of 1 × 10^−3^ s^−1^. Three dog-bone-shaped tensile specimens were selected, each with a gauge length of 30 mm, a width of 4 mm, and a thickness of 0.3 mm. The hardness was measured with a hardness tester with a load of 200 g and a holding time of 15 s. The samples for the tensile test were cut at the center of the Cu-Ag sheet and the samples for the hardness test were taken from the center of the Cu-Ag alloy.

## 3. Results

Figure 1a shows the aging treatment curves for the Cu-6 wt% Ag alloys. The samples were all aged at a constant temperature of 450 °C for 15 h with different cooling rates in the furnace, which were furnace cooling, 28 °C/h, 21 °C/h, and 17 °C/h. The variation of conductivity with deformation for the Cu-Ag alloy sheets under different cooling rates showed a similar trend, that is, a continuous decrease as the deformation increased, as shown in Figure 1b. The rolling was performed on the two-high mill with a diameter of the roll of 80 mm, a rotational velocity of the roll of 0.2 r/s, and a reduction per press of about 0.1~0.2%. A larger deformation can lead to more defects, which will cause a greater scattering of electrons. At the same deformation, the Cu-Ag alloy cooled in the furnace showed higher conductivity.

Compared with the conductivity, different aging cooling rates caused the Cu-Ag alloy to show different trends in tensile strength, as shown in Figure 1c. The strength of the furnace-cooled samples continued to increase as the rolling reduction increased, reaching a maximum strength of 1091.45 MPa. Although the strength of the sheets that underwent aging treatment at lower aging cooling rates decreased by 99.3% with rolling reduction, the strength of the samples at lower cooling rates was still higher than that at furnace cooling before the reduction reached 99.3%. In Sakai’s research [29], the Cu-Ag alloy has shown the same tendency when undergoing a certain aging treatment. 

Figure 1d shows the relationship between the strength and conductivity of the Cu-Ag alloy sheets after aging treatment at different cooling rates. The Cu-Ag alloy sheet with a reduced aging cooling rate showed better strength when the conductivity was higher than 76% IACS. With the decrease in cooling rate, the conductivity of the Cu-Ag alloy sheet reached 82% IACS and the tensile strength reached 970 MPa, while the strength of the sample of furnace cooling was only 850 MPa at the same conductivity. When the conductivity of the alloy reached 75% IACS, the strength of the sample of furnace cooling could reach 1003 MPa, which is higher than that of the other alloy samples whose cooling rate was reduced.

**Figure 2:**
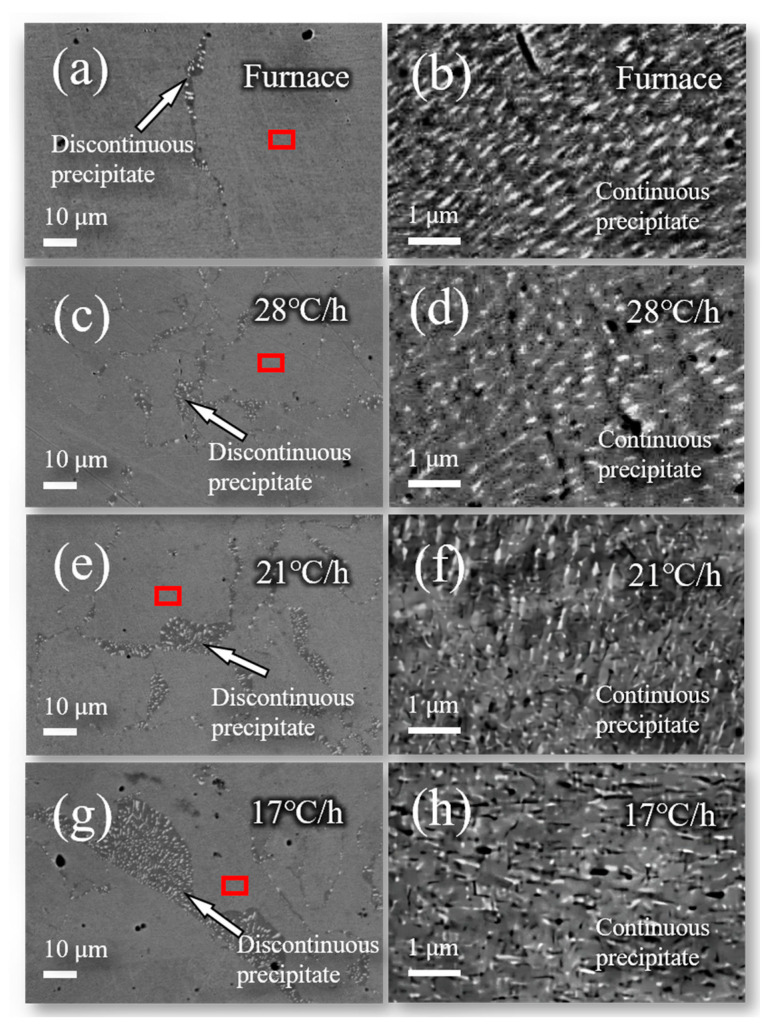
The BSE images of Cu-6 wt%Ag alloy after aging treatment at different cooling rates (**a**,**b**) furnace colling rate; (**c**,**d**) 28 °C/h; (**e**,**f**) 21 °C/h; (**g**,**h**) 17 °C/h. The arrows shown in (**a**,**c**,**e**,**g**) are the discontinuous precipitate, and the other areas are the continuous precipitate. The (**b**,**d**,**f**,**h**) is the magnification of the area indexed by the red box in (**a**,**c**,**e**,**g**).

In Figure 2, the morphology and distribution of the Ag precipitated phase (Ag β phase) of the Cu-6 wt%Ag alloy after aging treatment can be divided into two main regions. The first region is the discontinuous precipitated phase area, as shown in Figure 2a,c,e,g. Ag precipitates of approximately 0.5–2 μm in size are surrounded by a deeper color Cu matrix. These forms of Ag precipitates are shown as dots and strips. In Figure 2a,c,e,g, the discontinuous precipitated phase is banded and the region of the discontinuous precipitated phase gradually expands as the cooling rate decreases. When Cu-Ag was cooled at 17 °C/h, the discontinuous Ag precipitated phase had a bandwidth of 12 μm. The second region is the continuous precipitated phase, as shown in Figure 2b,d,f,h. The higher brightness of this part of the Cu matrix is due to the distribution of the nanoscale Ag precipitates. In Figure 2b, the nanoscale Ag precipitates cooled in the furnace are 50 nm in width and 70–460 nm in length. However, the size of these nanoscale Ag precipitates becomes smaller as the cooling rate decreases. The change between continuous and discontinuous precipitated phases of Ag implies that Ag atoms migrate during the aging treatment process. Differences in the microstructure of the Cu-Ag alloy after aging treatment will affect the trend of strength and electrical conductivity in the cold rolling process. It should be emphasized that the distribution of the Ag phase after heat treatment is an important factor in the properties of Cu-Ag alloys.

**Figure 3:**
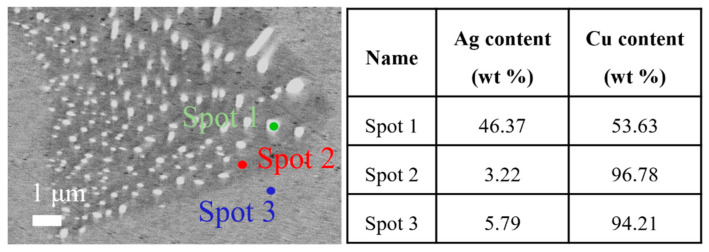
The EDS results of Cu-6 wt%Ag alloy heat-treated at 450 °C for 15 h and 17 °C/h cooling rate.

Figure 3 shows the EDS results of Cu-6 wt%Ag alloy aged at 450 °C for 15 h with the 17 °C/h cooling rate. Spot 1 is the Ag-rich phase, showing that the Ag content is 46.37%. Spot 2 is the Cu matrix portion of the discontinuous precipitation phase. Spot 3 is the area of nanoscale Ag precipitates with an Ag content of 5.79%. This discontinuous precipitation phase structure reduces the interfacial density of the Cu and Ag phases and contributes to improved electrical conductivity.

**Figure 4:**
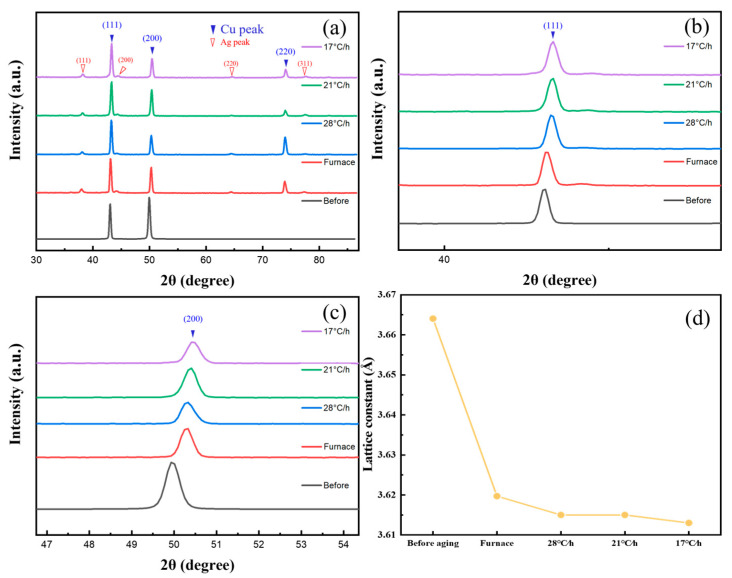
(**a**) XRD profiles of Cu-6 wt%Ag alloys before and after age treatment. (**b**) Local magnification of Cu (111) diffraction peak. (**c**) Local magnification of Cu (200) diffraction peak. (**d**) Lattice constants of Cu-6 wt%Ag alloy at different age cooling rates.

Figure 4a shows the XRD spectra of the Cu-Ag alloy before and after aging. Before the aging treatment, the Cu-Ag alloy was homogenized, the Ag atoms were solidly dissolved in the Cu lattice, and there are only diffraction peaks of Cu. After the aging treatment, a weaker Ag peak appears in the XRD spectrum due to the precipitation of the Ag phase during the aging process, and the diffraction peaks of the Cu matrix shift to the high angle (Figure 4b,c). The lattice parameters of Cu calculated using the Cu (111) peak in Figure 4a are shown in Figure 4d. The obvious decrease in the lattice constant of Cu after aging treatment is due to the precipitation of more Ag atoms from the Cu lattice, which reduces the distortion of the Cu lattice. This is similar to the result of Choi’s research [30]. The lattice constant of Cu decreases as the aging cooling rate slows down, which means there are more Ag atoms precipitating from the Cu matrix as the aging cooling rate decreases. According to the thermodynamics of precipitation, the slower the cooling rate is, the more time the Ag atoms have to move and precipitate. However, the content of Ag precipitates decreases in the continuous precipitated phase, which implies an increase in the discontinuous precipitated phase. This distribution of the Ag precipitated phase directly affects its mechanical and electrical properties.

**Figure 5:**
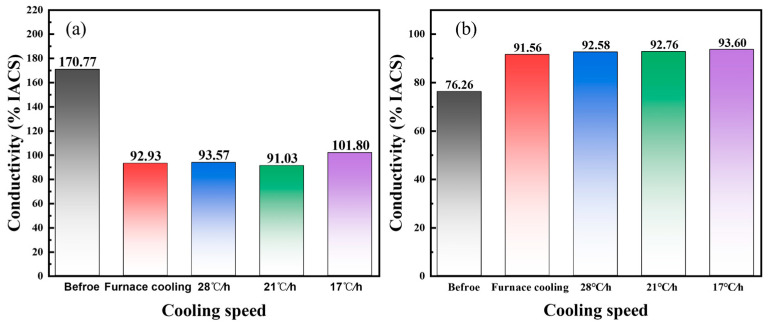
(**a**) The hardness and (**b**) electrical conductivity of Cu-6 wt%Ag alloy before and after aging treatment.

Figure 5a shows the hardness of the Cu-6 wt%Ag alloy before and after the aging treatment. Due to structural defects, such as vacancies, dislocations, and the distortion energy accumulated in the alloy by plastic deformation, the hardness of the alloy is as high as 170.77 HV. During aging treatment, although the Ag phase precipitates, the process of recovery, recrystallization, and grain growth occurs and the hardness of the alloy decreases, which is lower than 100 HV.

Figure 5b shows the variation of electrical conductivity of the Cu-6 wt%Ag alloy before and after the aging treatment. Since the conductivity is closely related to the point defects (vacancies, interstitial atoms, etc.), the deformed Cu-6 wt%Ag alloy before aging treatment shows a low electrical conductivity of 76.26% IACS. A significant increase in conductivity occurs due to the recovery, recrystallization, grain growth, and Ag phase precipitates from the matrix in the aging treatment. In addition, as the cooling rate of the aging treatment slows down, the discontinuous precipitation phase region in the Cu-Ag alloy broadens, the Ag precipitation phase size becomes larger, and the content of the Ag precipitation phase in the continuous precipitation phase region reduces, which reduces the two-phase interfaces and improves the electrical conductivity of the alloy. 

**Figure 6:**
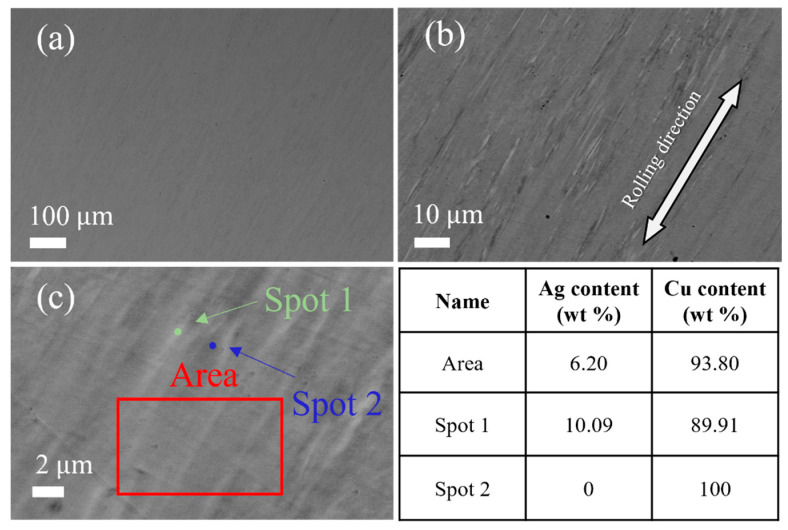
BSE image of (**a**,**b**) the cold-rolled Cu-6 wt%Ag alloy sheet with 99.3% reduction by furnace-cooled aging treatment and the EDX result of (**c**).

Figure 6 shows the microstructure of the cold-rolled Cu-6 wt%Ag alloy sheet with a 99.3% reduction followed by the furnace-cooled aging treatment. At low magnification (Figure 6a), the microstructure is very uniform, and no obvious contrast difference is observed. However, at high magnification (Figure 6b), the stripe-like Ag-rich phase, which stretches parallel to the rolling direction, is clearly seen. The size of this Ag-rich phase is refined, and its distribution is uniform. The EDX result shows that the average Ag content of the sheet is about 6% (the red box in Figure 6c), while the Ag content of the refined Ag-rich phase is about 10 wt% (Spot 1 in Figure 6c). The size of the Ag-rich phase ranges from 0.78 μm to 0.22 μm and the distribution becomes more uniform with increasing deformation. Since the evolution of the Ag-precipitated phases in Figure 2 is noteworthy, the shape of the Ag-rich phase evolved during the rolling process. Therefore, one key method for achieving high strength and high conductivity of Cu-Ag alloys is to control the morphology of Ag precipitates by using aging treatment before cold rolling. 

**Figure 7:**
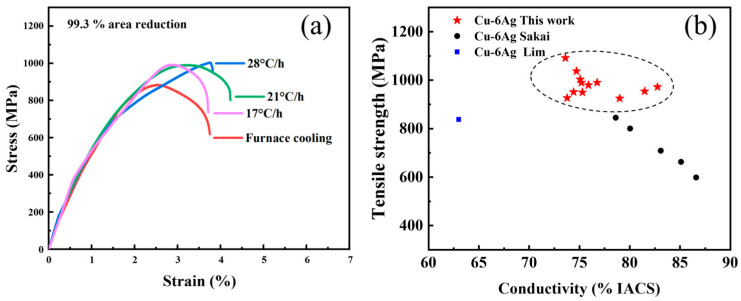
(**a**) Uniaxial tensile stress–strain curves of the Cu-6 wt%Ag sheet at a reduction of 99.3%. (**b**) Comparison of tensile strength and conductivity of Cu-6 wt%Ag sheets prepared using different methods [31,32].

Figure 7a shows the uniaxial tensile stress–strain curve of the Cu-6 wt%Ag sheet at a 99.3% reduction. Compared with the sample with furnace cooling, the tensile strength is significantly improved when the cooling rate decreases, which can exceed 1000 MPa at 28 °C/h. This high strength is attributed to the combined effects of precipitate strengthening and cold-work strengthening after severe deformation. Figure 7b shows the comparison of tensile strength and electrical conductivity of Cu-6 wt%Ag sheets prepared using different methods [31,32]. The Cu-6 wt%Ag sheets produced in this work show an excellent combination of high tensile strength and high electrical conductivity.

## 4. Discussion

From the above analysis, the Cu-Ag alloy sheets in this work show excellent comprehensive strength and electrical conductivity properties, which can be attributed to the dispersed continuous precipitates in the alloy. According to the literature, the precipitated phase is closely related to Ag atom diffusion at grain boundaries, and the nucleation and growth of the discontinuous precipitated phase are relatively easy for large-angle grains [27,33]. In Cu-Ag alloys with low Ag content, there are mainly large-angle grain boundaries, so Ag often forms discontinuous precipitated phases after aging treatment and it is difficult to obtain a large number of continuous precipitated phases [34,35]. The researchers found that the addition of Zr, Sc, and other elements can significantly inhibit the discontinuous precipitated phase and increase the continuous precipitated phase [16,19]. However, the addition of a third element significantly reduced the electrical conductivity of the alloy despite its high strength. In this work, the content of the continuous precipitated phase was increased by changing the aging process with different cooling rates. The continuous precipitated Ag phases were refined and elongated into the nanofiber after plastic deformation processing, which can further improve the strength of the alloy. However, the mechanism of the formation of the continuous precipitated phase at low Ag content still needs to be investigated further.

By considering the relationship between the cooling rate of aging treatment and the final properties, we put forward a method to regulate the comprehensive properties of alloys by adjusting the cooling rate of aging treatment. When higher tensile strength and relatively lower electrical conductivity (e.g., 1091 MPa, 73.6% IACS) are desired, the aging cooling rate needs to be increased so that more continuous precipitation occurs in the Cu-Ag alloy and large plastic deformation (e.g., deformation rate of 99.3%) is achieved. On the contrary, when higher electrical conductivity and lower tensile strength (e.g., 1003 MPa, 75% IACS) are expected, the cooling rate of aging treatment should be reduced to increase the discontinuous precipitated phase of the Cu-Ag alloy, and the alloy undergoes less plastic deformation (deformation rate 98.8%). This strategy lays a foundation for accurately controlling the comprehensive properties of Cu-Ag alloys in the future.

## 5. Conclusions

This work investigated the effect of the aging cooling rate on the microstructures and properties of Cu-6 wt%Ag alloy. The microstructural characterization showed that decreasing the aging cooling rate can affect the migration of Ag atoms, which increases the discontinuous precipitation phase and decreases the continuous precipitation phase of Ag. After severe cold rolling, the grains of Cu-Ag alloy and Ag precipitates were refined and the dislocation density was largely increased, which further improved the strength of the alloy. By controlling the cooling rate of the aging process and the rolling deformation, the Cu-6 wt%Ag alloy sheet (28 °C/h) can achieve an electrical conductivity of 75% IACS and a tensile strength of 1003 MPa. The Cu-Ag alloy sheets produced by this method are expected to serve as key conductors in water-cooled high-field magnets.

## Data Availability

The data supporting the reported results of this study can be made available from the corresponding author, upon request.
